# Effect of transcranial magnetic stimulation in combination with citalopram on patients with post-stroke depression

**DOI:** 10.3389/fnhum.2022.962231

**Published:** 2022-10-07

**Authors:** Zhen Zhu, Hao-Xuan Zhu, Shao-Wei Jing, Xia-Zhen Li, Xiao-Yan Yang, Tu-Nan Luo, Shuai Ye, Xiao-Chun Ouyang, Wei-Wei Song

**Affiliations:** ^1^Rehabilitation Medicine Department, The 908th Hospital of Chinese People's Liberation Army Joint Logistic Support Force, Nanchang, China; ^2^Department of Neurology, The 908th Hospital of Chinese People's Liberation Army Joint Logistic Support Force, Nanchang, China; ^3^Department of Neurology, Fuzong Clinical Medical College of Fujian Medical University (900 Hospital of the Joint Logistics Team), Fuzhou, China

**Keywords:** transcranial magnetic stimulation, citalopram, post-stroke depression, cognitive function, neuropsychological score

## Abstract

**Background:**

Amelioration of depression in patients with post-stroke depression (PSD) remains challenging.

**Objective:**

The primary vision was to explore the effect of transcranial magnetic stimulation (TMS) in combination with citalopram on patients with PSD.

**Methods:**

One hundred eligible patients who were diagnosed with PSD were recruited and randomly assigned to the control group (*n* = 50) or the TMS group (*n* = 50). The controls were given citalopram (10 mg/d for consecutive 8 weeks), while, in addition to citalopram, patients in the TMS group were also given TMS at 5 Hz once a workday for 8 weeks. The primary outcome was patient depression status as reflected by 17-item Hamilton Rating Scale for Depression (HAMD-17) score, and the secondary outcome was patient neuropsychological score determined by Mini-Mental State Examination (MMSE) and Wisconsin Card Sorting Test (WCST).

**Results:**

Patients treated with TMS in combination with citalopram had a drastic decrease in HAMD-17 score during treatment. Bigger changes in HAMD-17 score between baseline and 2 weeks as well as between baseline and 8 weeks in the TMS group were observed (*P* < 0.01). Patients in both groups had increased MMSE scores after treatment. Data of WCST revealed patients with TMS treatment completed more categories (*P* < 0.01) and had a lower RPP in comparison to patients in the control group (*P* < 0.0001). Additionally, TMS in combination with citalopram strikingly improved patients' MMSE scores when compared with those taking citalopram alone. Last, there was no striking difference in side effects between the two groups (*P* > 0.05).

**Conclusion:**

Our study found TMS in combination with citalopram is conducive to improving depression status and neuropsychological function, which holds great promise for treating PSD.

## Introduction

Stroke is the most frequent serious neurological disease, leading to a great number of deaths and disabilities, particularly in adults between 50 and 60 years old (Robinson and Jorge, 2016). Previous progress in diagnosis and neurosurgical treatment of stroke have strikingly diminished the mortality rate, leaving an elevated prevalence of stroke survivors (Ekker et al., 2018). Despite the elusive mechanism, previous studies have consistently shown a strong association between stroke and mental diseases (Cumming et al., 2016; Robinson and Jorge, 2016). Among those, post-stroke depression (PSD) is proven to be the most frequent condition in acute settings or rehabilitation units, with a prevalence of 33% (Hackett and Pickles, 2014). Not only does PSD cause depression, loss of interest, sleep disturbances, and cognitive and executive function impairment, but it could also delay rehabilitation and even increase the risk of suicide, which creates a heavy burden on patients and society (Mijajlovic et al., 2017). Moreover, PSD is likely to be most severe at an early stage; it is of great importance to palliate depressive symptoms as soon as possible when patients begin to seek help (Starkstein and Hayhow, 2019). Thus, enhancing the early diagnostic and therapeutic efficacy of PSD is highly imperative.

The current strategy to improve PSD mainly depends on several antidepressants, including the most commonly prescribed, paroxetine and citalopram (Villa et al., 2018). Although several small-size studies have indicated that citalopram exerted a remission of PSD, with an effectiveness of 47% (Starkstein and Hayhow, 2019), there is still a long way to go to find highly effective treatment to relieve depressive behaviors in PSD patients, especially for those who are at an early stage. As a non-invasive treatment, transcranial magnetic stimulation (TMS) has been widely used in rehabilitation units for the improvement of paralysis (Jo and Perez, 2020). It has also been recently proposed as a promising strategy for major depression (George et al., 2010). Patients with major depression find relief without adverse effects after being treated with TMS in combination with citalopram (Huang et al., 2012). In addition, TMS is also suggested to improve synaptic plasticity and neuropsychological functions in animal models and humans (Kaster et al., 2018; Pang and Shi, 2021), highlighting the therapeutic potential of TMS in the central nervous system. Previously, some researchers found some beneficial effects of TMS on patients with PSD, hastening the efficacy of antidepressants, however, it was limited in antidepressant-resident or late-stage patients. Larger scale studies are needed to determine the safety and efficacy of TMS based on antidepressants in PSD patients.

In the present study, we enrolled 100 PSD patients to determine the effect of TMS in combination with citalopram on patients with PSD. We found TMS in combination with citalopram significantly improved depression status and cognitive function in patients with PSD with no obvious adverse effects.

## Methods

### Subjects

A total of 100 first-episode PSD patients, from February 2021 to March 2022, were consecutively recruited. All patients were treated in the rehabilitation- and neurology departments of the 908th Hospital of the Chinese People's Liberation Army Joint Logistic Support Force. Patients were randomly assigned into two groups, the control group (*n* = 50) and the TMS group (*n* = 50). Notably, all patients in both groups received treatment of citalopram (10 mg per day for consecutive 8 weeks). We have obtained the consent for clinical information collection from patients, and this study was approved by the Institutional Review Board of the 908th Hospital of the Chinese People's Liberation Army Joint Logistic Support Force (reference number: 2022YYLL113).

### Inclusion and exclusion criteria

Patients with PSD were included if they complied with the following principles: (1) age over 18 years old; (2) clinical symptoms and imaging evidence showing they first suffered from ischemic stroke and PSD; (3) onset time < 7 day; (4) 17-item Hamilton Rating Scale for Depression (HAMD-17) between 7 and 24; (5) the National Institute of Health Stroke Scale (NIHSS) score was 1–15 when admitted; (6) patient's family signed the informed consent. Contrarily, patients were excluded if they met either of the following principles: (1) age below 18 years old; (2) patients didn't meet the diagnosis of PSD; (3) intellectual, language, or expression dysfunction; (4) a history of depression or anxiety.

### Treatment

Conventional treatments including hyperbaric oxygen treatment, blood pressure control, neurotrophic support treatment, and symptomatic treatment supplemented by acupuncture, massage, and rehabilitation training, were applied to all patients. As mentioned above, patients in the control group were given oral citalopram (Xian-Janssen Pharmaceutical Ltd, China) 10 mg per day for consecutive 8 weeks. Apart from this, patients in the TMS group also received repetitive TMS once a day for 5 workdays a week for 8 weeks. The experimental setting of TMS was according to the treatment recommendations of TMS for major depressive disorder (Perera et al., 2016). Briefly, the stimulation site was selected as the left dorsolateral prefrontal cortex (LDLPFC) at a magnetic stimulation frequency of 5 Hz, matching specifications of a prior study (Li et al., 2022). Before the probe test, an individual's level of movement threshold (MT) needed to be determined, which was defined as when the contralateral thumb abductor motor evoked potential exceeds 50 μV for at least 5 times in ten stimulations, the minimum stimulus intensity is MT. During probe treatment, patients were placed in a sitting or supine position. The magnetic stimulation intensity was 60% MT, and the interval was 56 s for every 4 s stimulation, and the total stimulation period was 20 min. Notably, 800 magnetic pulse stimulation was given every day.

### NIHSS

The NIHSS comprises 15 items of a neurologic examination stroke scale for evaluating patient status after acute cerebral infarction. The details regarding the 15 items are presented in previous literature (Kwah and Diong, 2014). A well-trained neurosurgeon was responsible for rating patient ability to answer and perform.

### HAMD-17

A HAMD-17 assessment was conducted according to a previous study (Zimmerman et al., 2013). In brief, the scale encompasses seventeen aspects, including sleep disorder, cognitive impairment, tardiness, and sense of hopelessness. The higher the total score is, the more severe the degree of depression is. According to the scores, depression severity is segmented into three statuses: mild depression (8–17), moderate depression (17–24), and severe depression (≥24). All patients received the HAMD-17 assessment at 0-, 2-, 4-, 6-, and 8-weeks after stroke.

### Mini-mental state examination

The Chinese version of Mini-Mental State Examination (MMSE) was performed to assess the cognitive function, which encompasses tests of attention, orientation, memory, language, visual-spatial skills, etc. The total score of MMSE is 30, and the cognitive function is reflected by the score.

### Wisconsin card sorting test

The Wisconsin Card Sorting Test (WCST) was used for the neuropsychological function of patients according to a previous method (Huang et al., 2012). Subjects were required to sort 48 cards presented on the computer, which are categorized into three conditions: color, shape, and number. Patients were required to change one sorting to another one if they had consecutively completed six correct tasks. The end of WCST was defined as the correct completion of all six categories using 48 cards. Several parameters, including the total number of errors, total trials, correct trials, etc., were recorded and assessed blindly.

### Statistical analysis

All data in the present study were analyzed by SPSS 26.0 and graphed by Graphed Prism. Except for those especially noted, data were presented as mean ± SD. An independent *t*-test was used to determine differences between the two groups if the data complied with the normality examined by Kolmogorov–Smirnov test. Otherwise, the Mann-Whitney U test was applied. Comparisons between two categorical groups were performed by a chi-square test. A paired *t*-test was conducted to determine the difference between before and after treatment in the same group. Differences between the two groups at different time points were determined by repetitive measuring tests. The sample size was calculated using an online model (http://www.powerandsamplesize.com/). A *P* < 0.05 was considered statistically significant.

## Results

### Patient characteristics

As is shown in Table 1, a total of 100 patients with PSD were enrolled in our study, the control group (*n* = 50) and the TMS group (*n* = 50). There was no significant difference in age, gender, BMI, the incidence of hypertension, or rate of diabetes between the two groups (*P* > 0.05). Also, the onset time of PSD did not differ in the two groups and neither did education level (*P* > 0.05). Notably, cognitive function, as reflected by MMSE, also didn't differ between the two groups (*P* > 0.05). Additionally, patients in the control group bore a close resemblance to the TMS group in depression severity indicated by HAMD-17 (*P* > 0.05).

**Table 1:** Demographic characteristics.

**Variable**	**Control group**	**TMS group**	**Statistics**	***P*-value**
No. of patients	50	50		
Age (years)	48.13 ± 13.42	48.25 ± 12.82	*t* = 0.0457	0.964
Male/female	24 (26)	27 (23)	χ^2^ = 0.360	0.5485
Onset time of PSD	4.23 ± 2.25	4.15 ± 1.82	*t* = 0.196	0.845
BMI (kg/m^2^)	23.11 ± 2.41	23.28 ± 1.34	*t* = 0.436	0.664
Education level (years)	11.34 ± 3.25	11.84 ± 2.97	*t* = 0.803	0.424
Hypertension (%)	10 (20)	9 (22.5)	χ^2^ = 0.0650	0.799
Diabetes (%)	5 (10)	6 (12)	χ^2^ = 0.102	0.749
MMSE (at admission)	25.34 ± 3.21	25.49 ± 2.29	*t* = 0.270	0.789
HAMD-17 (at admission)	24.54 ± 2.27	24.40 ± 3.01	*t* = 0.793	0.782

BMI, body mass index; Control group, citalopram + sham TMS; TMS group, citalopram + TMS.

### Effect of TMS in combination with citalopram on depression status

**Figure 1:**
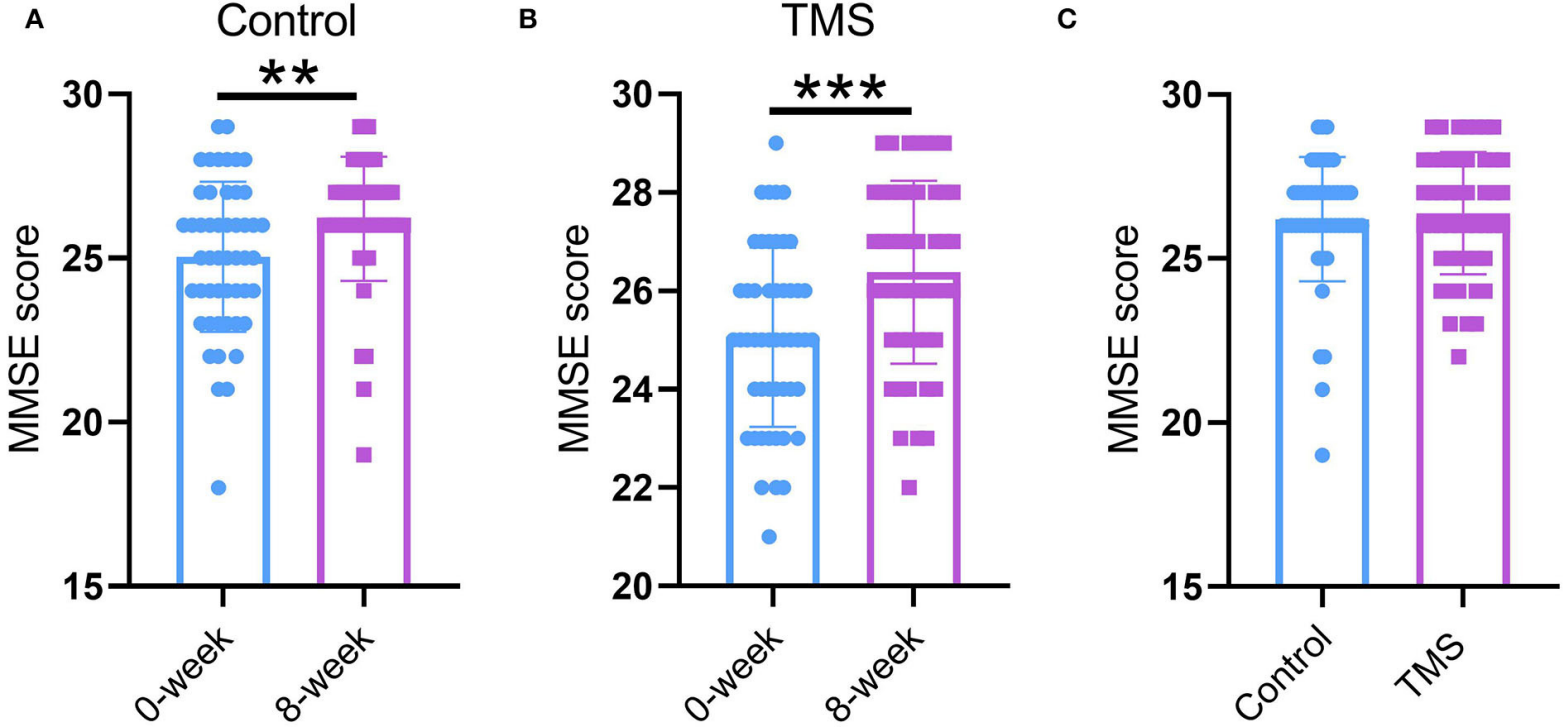
Effect of TMS in combination with citalopram on depression status. **(A)** HAMD-17 score from baseline throughout 8 weeks of treatment. **(B)** Changes of HAMD-17 score between baseline and 2 weeks. **(C)** Changes of HAMD-17 score between baseline and 8 weeks. ** *P* < 0.01, *** *P* < 0.001. *N* = 50 per group. The error bars present the SD.

To interrogate the effect of TMS in combination with citalopram on depression severity (primary outcome), we conducted HAMD-17 assessment at 0-, 2-, 4-, 6-, and 8-weeks for all patients with PSD. As mentioned above, there was no striking difference in HAMD-17 between groups at admission. In general, we found a more dramatic decrease in HAMD-17 score in the TMS group than in the control group during the 8-week treatment (*P* < 0.0001, Figure 1A). Indeed, the sub-analysis showed that changes in HAMD-17 score between baseline and 2 weeks in the TMS group were much higher than that in the control group (*P* < 0.01, Figure 1B). In addition, we also found patients in the control group had a higher HAMD-17 score than that of the TMS group (*P* < 0.01, Figure 1C), indicating a severe depression status, whereas changes in HAMD-17 score between baseline and 8 weeks in TMS group was higher than that in the control group (*P* < 0.01).

### Effect of TMS in combination with citalopram on neuropsychological score

**Figure 2:**
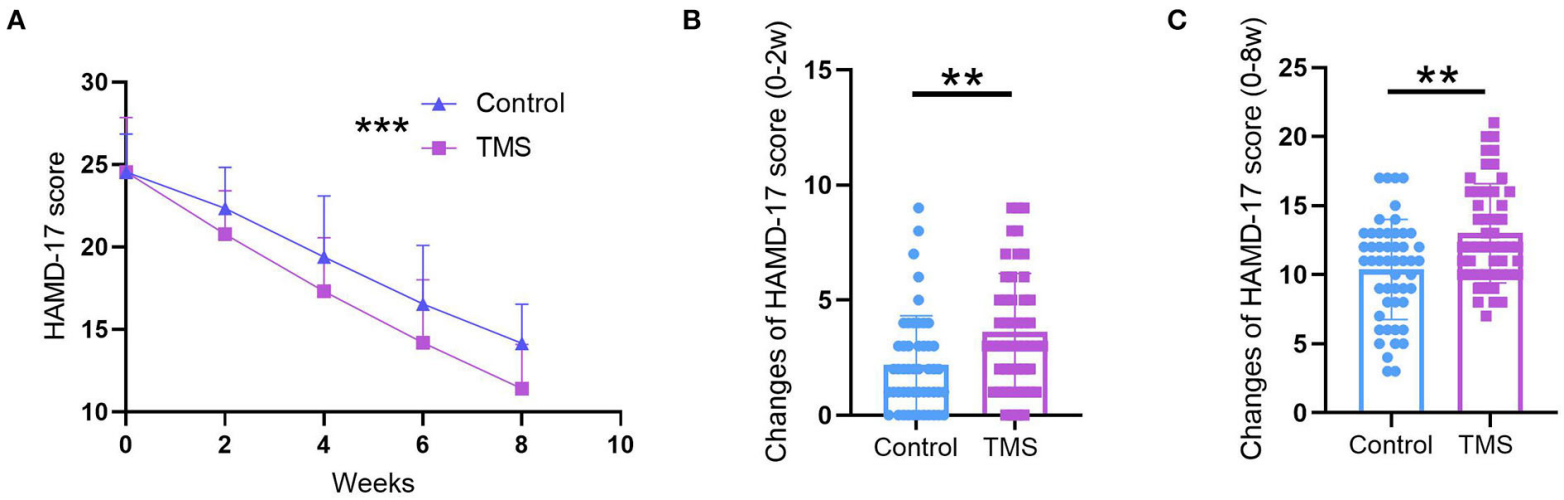
Effect of TMS in combination with citalopram on MMSE score. **(A)** Comparison of MMSE scores between 0-weeks and 8-weeks in the control group. **(B)** Comparison of MMSE score between 0-weeks and 8-weeks in TMS group. **(C)** Comparison of MMSE score between the control group and TMS group at 8-weeks. ** *P* < 0.01, *** *P* < 0.001. *N* = 50 per group. The error bars present the SD.

We also performed an MMSE assessment and WCST to investigate the neuropsychological status (second outcome). The data showed MMSE scores in both the control and TMS group were increased after treatment (*P* < 0.05 for the control group; *P* < 0.001 for the TMS group; Figures 2A,B), whereas there was no remarkable difference in MMSE scores between the two groups (*P* > 0.05, Figure 2C). Additionally, in the WSCT task, patients in two groups had improvements in CC, RFPP, and RPP. Moreover, patients with TMS treatment completed more categories (*P* < 0.01, Table 2) and had a lower RPP in comparison with patients in the control group (*P* < 0.0001, Table 2). Lastly, we also found TMS in combination with citalopram strikingly improved the NIHSS and MMSE scores in comparison with citalopram alone (Table 3).

**Table 2:** Results of WCST.

**Variable**	**Control group**		**TMS group**	
	**Baseline**	**Week 8**	**Baseline**	**Week 8**
CC	2.41 ± 0.72	2.92 ± 0.78*	2.43 ± 0.91	3.46 ± 1.12
RFPP	0.33 ± 0.22	0.48 ± 0.12^†^	0.34 ± 0.11	0.53 ± 0.34^†^
RPP	0.53 ± 0.11	0.31 ± 0.10^†^	0.55 ± 0.12	0.21 ± 0.07^§^

Control group, citalopram + sham TMS; TMS group, citalopram + TMS. CC, categories completed; RFPP, percent conceptual level responses percentage; PRP, perseverative responses percentage. vs. baseline, ^*^P < 0.01; vs. baseline,^†^P < 0.001; vs. week 8,^‡^P < 0.01; vs. week, ^§^P < 0.0001.

**Table 3:** Results of MMSE and NIHSS score.

**Variable**	**Control group**		**TMS group**	
	**Baseline**	**Week 8**	**Baseline**	**Week 8**
MMSE	25.34 ± 3.11	26.33 ± 1.05*	25.25 ± 3.22	28.25 ± 1.23^§^
NIHSS	10.31 ± 2.22	4.48 ± 1.12^†^	10.28 ± 2.11	3.23 ± 1.34

Control group, citalopram + sham TMS; TMS group, citalopram + TMS. MMSE, Mini-mental State Examination; NIHSS, National Institute of Health Stroke Scale (NIHSS). vs. baseline, ^*^P < 0.01; vs. baseline,^†^P < 0.001; vs. week 8,^‡^P < 0.001; vs. week 8, ^§^P < 0.0001.

### The impact of TMS in combination with citalopram on the occurrence of side effects

Despite a high incidence of headache, nausea, and dizziness in both groups, there was no striking difference in those side effects between the two groups (*P* > 0.05, Table 4). We reasoned the high incidence of headache, nausea, and dizziness resulted from the stroke itself.

**Table 4:** Occurrence of side effects during treatment.

**Variable**	**Control group**	**TMS group**	**Statistics**	***P*-value**
Headache (%)	40 (80)	42 (84)	χ^2^ = 0.271	0.603
Nausea (%)	11 (22)	10 (20)	χ^2^ = 0.219	0.640
Dizziness (%)	35 (70)	39 (78)	χ^2^ = 1.372	0.242

Control group, citalopram + sham TMS; TMS group, citalopram + TMS.

## Discussion

In the present study, we investigated the safety and efficacy of TMS in combination with citalopram in patients with PSD, with merely citalopram administration as a control. Dramatically, we found TMS in combination with citalopram significantly improved depression status in patients with PSD with no obvious adverse effects. In addition, our study also revealed TMS in combination with citalopram could exert an improving function in neuropsychological tests, including MMSE and WCST.

PSD has been proven to pose a high risk to individuals who survive stroke for causing depression, loss of interest, and delaying rehabilitation, even elevating the possibility of suicide, which creates a grave burden for families and society (Robinson and Jorge, 2016). Disease-mortifying treatment, such as citalopram, has been widely utilized to alleviate symptoms of PSD without dissatisfying outcomes, indicating a single treatment might not be enough to obtain positive outcomes. Enhancing the efficacy of disease-mortifying treatment is the goal for doctors to pursue. TMS has been widely utilized to promote recovery of motor functions after spinal cord injury (Jo and Perez, 2020). Recently, TMS has also been proposed as a promising strategy for improving depression status (Huang et al., 2012). A recent study on patients with major depression revealed that TMS in combination with citalopram bore an improving function in relieving depression symptoms (Huang et al., 2012). The primary purpose was to investigate the effect of TMS in combination with citalopram on PSD patients. Dramatically, we found a remarkable improvement in depression status after treatment of TMS in combination with citalopram, as reflected by HAMD-17 scores. As mentioned in the introduction, relieving symptoms at an early stage of PSD holds great importance during treatment as a whole (Mijajlovic et al., 2017). Sub-analysis found that TMS enhanced symptom improvement in the first 2 weeks when regularly treated in an early period of PSD, indicating TMS in combination with citalopram improved PSD at an early stage. Although we found that patients have some discomforts, such as headache, nausea, and dizziness, those discomforts might derive from the stroke itself. Taken together, our findings are consistent with previous studies that repetitive TMS is an effective and safe treatment for PSD (Li et al., 2022; Shen et al., 2022).

The brain dysfunction of depressive patients is mainly related to the abnormal interaction between the cortex and subcortical nuclei (Villa et al., 2018). LDLPFC is involved in positive emotion regulation, while the right DLPFC (RDLPFC) is involved in negative emotion regulation. In general, DLPFC usually presents a weakened function in depressive patients, while the right DLPFC function was significantly enhanced (Fitzgerald et al., 2006). Thus, it is theoretically beneficial to alleviate the symptoms of patients with depression by adjusting the function of DLPFC on both sides of the patient's brain. In the present study, we selected LDLPFC as the stimulation site for TMS that intensified the brain function and added the evidence that this action site indeed is a therapeutic target for attenuating PSD. Another critical concern that should also be noted is that the frequency of TMS was 5 Hz in our study. High frequent stimulation of TMS for LDLPFC and low frequency for RDLPFC are preferred. Previously a systematic review from China found that 10 Hz could evoke the left-brain function and palliate the PSD (Shao et al., 2021). Our study found that 5 Hz was enough to obtain those protective effects, which is also supported by another study (Li et al., 2022).

This leaves the question of how does TMS in combination with citalopram improve the symptoms of PSD. Previous analysis revealed that PSD is related to the disrupted neurotransmitter metabolism, particularly for decreased 5-HT and norepinephrine (NE) neurotransmitter contents in lesioned regions (Liu et al., 2022). Citalopram is a kind of 5-HT reuptake inhibitor that reduces 5-HT reuptake by neurons, thereby producing antidepressant effects. Dopamine (DA) is one of the most important neurotransmitters in the NE system. DA contents in the brain were proved to be decreased after stroke, as confirmed by animal and clinical research (Grace, 2016; Liu et al., 2022). Considering the provoking effects of TMS in the cortex, we speculate that TMS might activate the cingulate gyrus, putamen, hippocampus, and thalamus through the frontal to subcortical nucleus neural circuits, and activate the contralateral region through the corpus callosum to enhance the dopamine (DA) release of the striatum and mesolimbic system (Fitzgerald et al., 2006). This notion is also supported by a recent study that TMS effectively promotes the synthesis and release of DA in patients with PSD (Liu et al., 2022). In those regards, TMS in combination with citalopram holds more therapeutic value for treating PSD in comparison with only citalopram administration.

In addition, we also conducted the neuropsychological assessment, including MMSE and WSCT, to explore the cognitive function and working memory. A previous study has revealed that PSD was always companied by disrupted cognitive function and working memory (Price and Duman, 2020). Our findings uncovered that TMS in combination with citalopram improved those disruptions, which might result from improved depression status or the recovery of stroke.

There are some limitations in our study. First, we don't provide the direct evidence to prove the beneficial role of TMS in ameliorating the depressive symptoms of PSD. Second, other confounders, such as placebo effect or other medications, might also bring the additive outcomes. Last, we only included the HAMD-17 scale to evaluate the primary outcome, which might not fully reflect the depressive status.

## Conclusion

Our study found TMS in combination with citalopram is conducive to improving depression status and neuropsychological function, which holds great promise for treating PSD.

## Data availability statement

The original contributions presented in the study are included in the article/supplementary material, further inquiries can be directed to the corresponding author.

## Ethics statement

The studies involving human participants were reviewed and approved by the Institutional Review Board of the 908th Hospital of the Chinese People's Liberation Army Joint Logistic Support Force. The patients/participants provided their written informed consent to participate in this study.

## Author contributions

ZZ, W-WS, and X-CO conceived and designed the project. ZZ, SY, and H-XZ collected the data. ZZ, H-XZ, S-WJ, X-ZL, X-YY, and T-NL analyzed data. ZZ and W-WS wrote the manuscript. All authors contributed to the article and approved the submitted version.

## Funding

This study was supported by the 908th Hospital of the Chinese People's Liberation Army Joint Logistic Support Force (Funding is to W-WS). The funding had no role in the present study.

## Conflict of interest

The authors declare that the research was conducted in the absence of any commercial or financial relationships that could be construed as a potential conflict of interest.

## Publisher's note

All claims expressed in this article are solely those of the authors and do not necessarily represent those of their affiliated organizations, or those of the publisher, the editors and the reviewers. Any product that may be evaluated in this article, or claim that may be made by its manufacturer, is not guaranteed or endorsed by the publisher.
